# Cellular and Subcellular Localisation of Kv4-Associated KChIP Proteins in the Rat Cerebellum

**DOI:** 10.3390/ijms21176403

**Published:** 2020-09-03

**Authors:** Rocío Alfaro-Ruíz, Carolina Aguado, Alejandro Martín-Belmonte, Ana Esther Moreno-Martínez, Rafael Luján

**Affiliations:** Synaptic Structure Laboratory, Instituto de Investigación en Discapacidades Neurológicas (IDINE), Dept. Ciencias Médicas, Facultad de Medicina, Universidad Castilla-La Mancha, Campus Biosanitario, C/Almansa 14, 02008 Albacete, Spain; Rocio.Alfaro@uclm.es (R.A.-R.); Carolina.Aguado@uclm.es (C.A.); Alejandro.Martin@uclm.es (A.M.-B.); AnaEsther.Moreno@uclm.es (A.E.M.-M.)

**Keywords:** cerebellum, potassium channel, KChIP proteins, electron microscopy, immunohistochemistry

## Abstract

The K^+^ channel interacting proteins (KChIPs) are a family of cytosolic proteins that interact with Kv4 channels, leading to higher current density, modulation of channel inactivation and faster recovery from inactivation. Using immunohistochemical techniques at the light and electron microscopic level combined with quantitative analysis, we investigated the cellular and subcellular localisation of KChIP3 and KChIP4 to compare their distribution patterns with those for Kv4.2 and Kv4.3 in the cerebellar cortex. Immunohistochemistry at the light microscopic level demonstrated that KChIP3, KChIP4, Kv4.2 and Kv4.3 proteins were widely expressed in the cerebellum, with mostly overlapping patterns. Immunoelectron microscopic techniques showed that KChIP3, KChIP4, Kv4.2 and Kv4.3 shared virtually the same somato-dendritic domains of Purkinje cells and granule cells. Application of quantitative approaches showed that KChIP3 and KChIP4 were mainly membrane-associated, but also present at cytoplasmic sites close to the plasma membrane, in dendritic spines and shafts of Purkinje cells (PCs) and dendrites of granule cells (GCs). Similarly, immunoparticles for Kv4.2 and Kv4.3 were observed along the plasma membrane and at intracellular sites in the same neuron populations. In addition to the preferential postsynaptic distribution, KChIPs and Kv4 were also distributed presynaptically in parallel fibres and mossy fibres. Immunoparticles for KChIP3, KChIP4 and Kv4.3 were detected in parallel fibres, and KChIP3, KChIP4, Kv4.2 and Kv4.3 were found in parallel fibres, indicating that composition of KChIP and Kv4 seems to be input-dependent. Together, our findings unravelled previously uncharacterised KChIP and Kv4 subcellular localisation patterns in neurons, revealed that KChIP have additional Kv4-unrelated functions in the cerebellum and support the formation of macromolecular complexes between KChIP3 and KChIP4 with heterotetrameric Kv4.2/Kv4.3 channels.

## 1. Introduction

The cerebellum is a foliated structure formed by the vermis and two symmetric hemispheres [1] that plays a major role in the fine motor control, maintenance of balance and posture, but also contributes to perception, memory and cognition [2,3]. Although the interplay among all cerebellar cells is central to fulfil those functions, Purkinje cells and granule cells are of key importance. GCs integrate sensory information arriving through the mossy fibres and thereby modulate the activity of PCs, which generate the sole output from the cerebellar cortex [2]. The function of cerebellar neurons depends on their synchronous activity, which is controlled by organised excitatory and inhibitory inputs to dendritic spines and shafts, as well as by intrinsic conductance that cause them to fire determined by ion channels’ activation [4].

Voltage-gated potassium (Kv) channels are involved in controlling the excitability and function of cerebellar neurons [5]. Particularly, Kv4 channels underlie somato-dendritic rapidly inactivating currents, known as A-type currents. Formed by three Kv4 α subunits (Kv4.1, Kv4.2 and Kv4.3), these currents play significant roles in dendritic excitability [6,7,8]. In addition to membrane-spanning dipeptidyl aminopeptidase-like proteins (DPPs) and Alzheimer’s disease-associated presenilin-2 [9,10], native neuronal Kv4 channels associate with cytoplasmic Kv channel interacting proteins (KChIPs) [11,12]. KChIPs bind to the N-terminal domain of Kv4 α subunits, and are classified within the family of Ca^2+^-binding proteins [11,13]. Four subtypes of KChIP proteins (KChIP1–KChIP4) have been cloned [10,11,13,14], all of which exist in alternately spliced variants [15]. In the brain, KChIPs regulate biophysical, biochemical and cell biological properties of Kv4 channels. For example, KChIP increases the surface expression and the current amplitude of Kv4 [16,17], slows the turnover rate of Kv4 protein and also slows the inactivation kinetics and speeds the rate of recovery from inactivation of Kv4 channels [8,11,16]. Consistent with their function in modulating K^+^ channels, deletion of Kv4.2 channel eliminates expression of its associated KChIP proteins [18].

In situ hybridisation studies have established that KChIP1, KChIP2, KChIP3 and KChIP4 proteins are differentially and widely expressed in the brain [14,19,20,21], where they associate with Kv4 channels. The pattern of KChIP1–4 association with Kv4.2 or Kv4.3 varies depending on the brain region and cell type [20,22]. The cerebellum exemplifies a brain region where the four KChIP proteins are expressed [14,19,20,21], and also shows high levels of both Kv4.2 and Kv4.3 subunits [23]. At the cellular level, KChIP subunits are expressed in PCs and GCs showing overlapping patterns and remarkable correspondence with the cellular expression of Kv4.2 and Kv4.3 [11,19,20,23]. As far as we know, detailed information regarding how these four proteins are organised in different subcellular compartments of cerebellar neurons is still missing. Thus, to investigate the subcellular localisation of KChIPs and their spatial association with Kv4 α subunits in cerebellar neurons, we combined high-resolution immunoelectron microscopy approaches with quantitative analyses. Our data suggest that A-type K+ channels containing Kv4.2, Kv4.3, KChIP3 and KChIP4 regulate postsynaptic excitability at different neurons in the cerebellum.

## 2. Results

### 2.1. Regional Distribution of Kv4 and KChIPs in the Cerebellum

Using subtype-specific monoclonal and polyclonal antibodies, we recently found that Kv4.2 and Kv4.3 subunits exhibit differential expression and distribution patterns in rodent hippocampus [24]. Here, using monoclonal antibodies against auxiliary KChIP3 and KChIP4 subunits, together with the antibodies against Kv4.2 and Kv4.3 subunits, we have now investigated the distribution of these molecules in the cerebellum using light microscopy immunohistochemical techniques.

**Figure 1:**
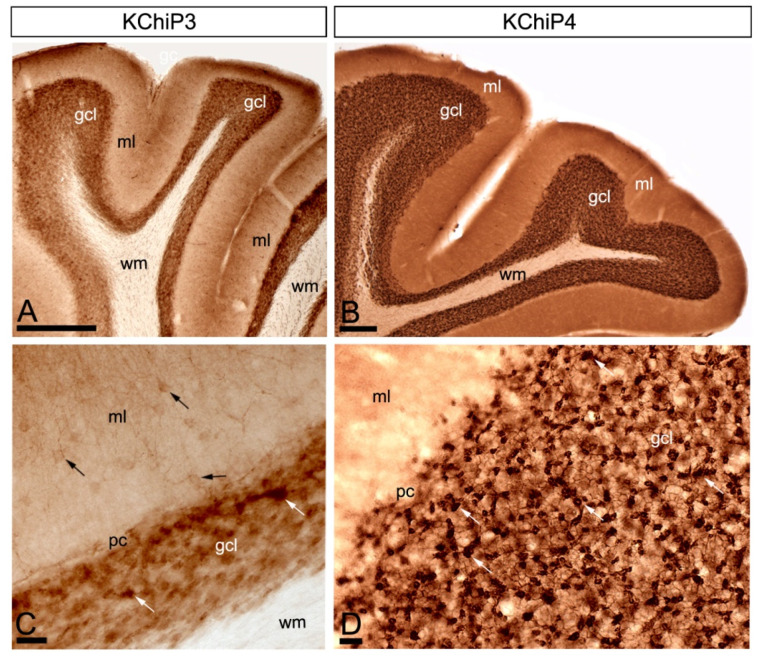
Regional and cellular distribution of Kv channel interacting proteins (KChIPs) subunits KChIP3 and KChIP4 in the cerebellum. (**A**–**D**) Immunoreactivity for KChIP3 and KChIP4 in the cerebellar cortex using a pre-embedding immunoperoxidase method at the light microscopic level. Parasagittal photomicrographs of the cerebellar cortex. Immunoreactivity for both KChIP3 and KChIP4 was widely distributed in the cerebellar cortex with mostly overlapping labelling patterns. Although with some differences in intensity of labelling, strong immunoreactivity for KChIP3 and KChIP4 was found in the granule cell layer (gcl) and weaker in the molecular layer (ml) and the white matter (wm) was devoid of any staining. In the molecular layer, KChIP3 and KChIP4 was mostly neuropilar and KChIP3 labelling was also detected in cell bodies and dendrites of stellate and basket cells (black arrows). In the granule cell layer, KChIP3 and KChIP4 particularly concentrated in glomeruli (white arrows) and surrounding GCs. Scale bars: (**A**), 50 µm; (**B**), 100 µm; (**C**,**D**), 25 µm.

Immunoreactivity for KChIP3 and KChIP4 was widely distributed in the cerebellar cortex, showing distribution patterns that mostly overlapped in the granule cell layer and molecular layer (Figure 1A–D). Immunolabelling for both subunits was significantly stronger in the granule cell layer compared to the molecular layer, and very weak in the white matter (Figure 1A–D). In the molecular layer, immunolabelling for KChIP3 and KChIP4 was mostly neuropilar, but KChIP3 labelling was also observed in cell bodies and dendrites of stellate and basket cells (Figure 1C,D). In the granule cell layer, immunoreactivity for KChIP3 and KChIP4 was particularly concentrated surrounding GCs and in glomeruli (Figure 1C,D). The high level of expression of KChIP3 and KChIP4 protein in the granule cell layer of the cerebellar cortex suggests a somato-dendritic localisation of both subunits in GCs.

**Figure 2:**
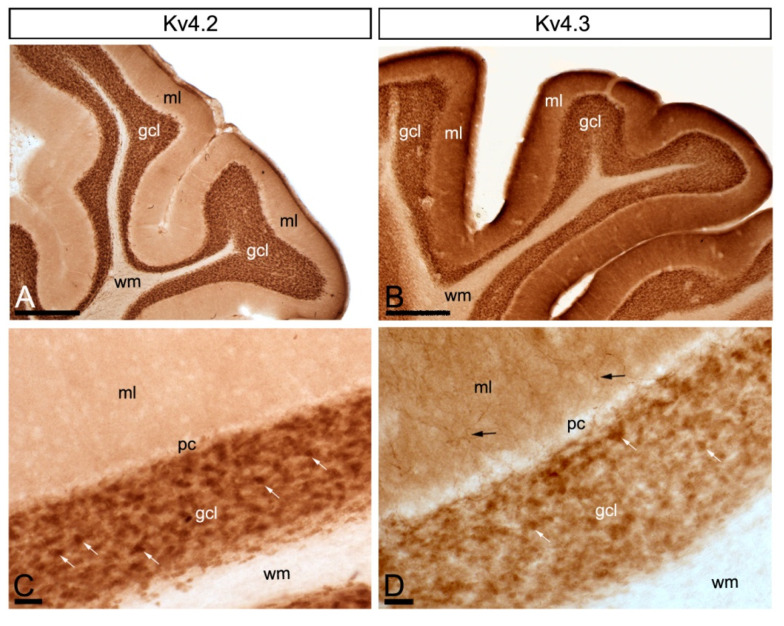
Regional and cellular distribution of voltage-gated potassium (Kv) channel subunits Kv4.2 and Kv4.3 in the cerebellum. (**A**–**D**) Immunoreactivity for Kv4.2 and Kv4.3 in the rat cerebellar cortex using a pre-embedding immunoperoxidase method at the light microscopic level. Parasagittal photomicrographs of the cerebellar cortex. The strongest immunoreactivity for Kv4.2 and Kv4.3 was found in the granule cell layer (gcl). Strong immunoreactivity for Kv4.3 was also observed in the molecular layer (ml), but weaker for Kv4.2. The white matter (wm) was always devoid of any immunolabelling. Immunoreactivity for Kv4.2 and Kv4.3 in the molecular layer was mostly neuropilar, but Kv4.3 labelling was also detected in cell bodies and dendrites of basket cells (black arrows). In the granule cell layer, Kv4.2 and Kv4.3 immunolabelling particularly concentrated in glomeruli (white arrows) and surrounding GCs. Scale bars: (**A**,**B**), 50 µm; (**C**,**D**), 25 µm.

Similar to KChIP subunits, immunoreactivity for Kv4.2 and Kv4.3 was widely distributed in the cerebellar cortex, showing overlapping labelling patterns (Figure 2A–D). Immunolabelling for Kv4.2 and Kv4.3 was strongest in the granule cell layer, mainly associated with glomeruli and with GCs (Figure 2C,D). In contrast, immunoreactivity for Kv4.2 and Kv4.3 was lower in the molecular layer, but while labelling for Kv4.2 was detected in the neuropil, labelling for Kv4.3 was observed both in the soma and dendrites of interneurons and in the neuropil (Figure 2C,D).

Overall, this remarkably similar pattern of immunolabelling for KChIP3, KChIP4, Kv4.2 and Kv4.3, particularly in the granule cell layer, raises the possibility that these molecules may co-assemble in heteromeric Kv channels in GCs.

### 2.2. Subcellular Localisation of KChIP3 in the Cerebellum

To investigate the subcellular localisation of KChIP protein subunits in cerebellar neurons with high spatial resolution, we performed electron microscopic studies using the pre-embedding immunogold technique combined with quantitative analyses in the cerebellar cortex. Cerebellar samples used for the descriptions of results below belong to folia III to V of the cerebellum, thus avoiding possible variations due to differences in composition and connections from folia I to X.

**Figure 3:**
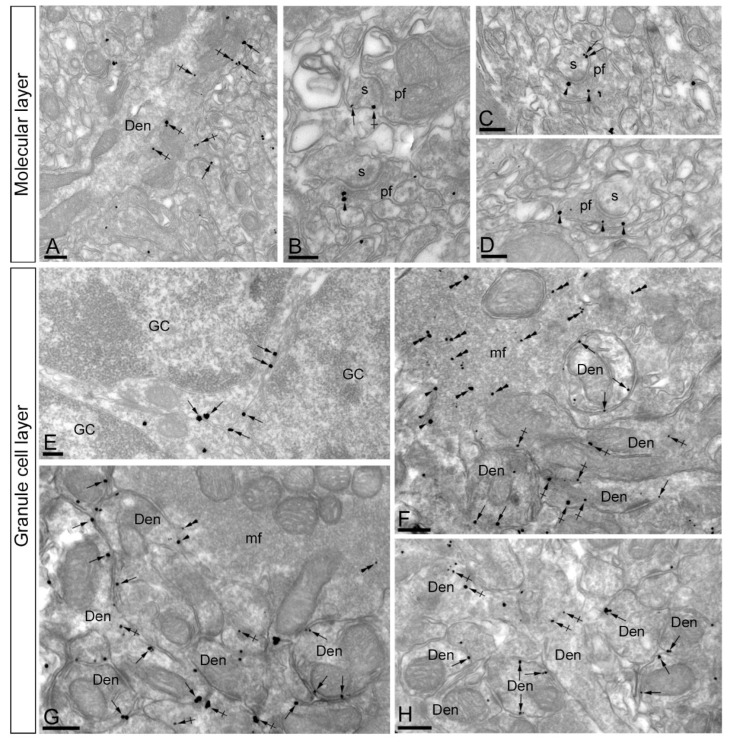
Subcellular localisation of Kv channel interacting protein (KChIP) subunit KChIP3 in the cerebellum. Electron micrographs showing immunoparticles for KChIP3 in the cerebellar cortex, as detected using the pre-embedding immunogold technique. (**A**–**D**) In dendritic shafts (Den) and spines (s) of PCs, located in the molecular layer, KChIP3 immunoparticles were observed postsynaptically along the extrasynaptic plasma membrane (arrows). Less frequently, KChIP3 immunoparticles were observed intracellularly (crossed arrows). Presynaptically, KChIP3 immunoparticles were detected along the extrasynaptic membrane and at intracellular sites (arrowheads) of parallel fibres (pf). (**E**–**H**) In the granule cell layer, KChIP3 immunoparticles were observed along the somatic plasma membrane (arrows) and dendrites (Den, arrows) and intracellular sites (crossed arrows) of granule cells (GC). Presynaptically, KChIP3 immunoparticles were observed along the plasma membrane (arrowheads) of mossy fibre axon terminals (mf), but most of them were localised intracellularly (double arrowheads) associated with cytoplasmic membranes. Scale bars: (**A**–**C**), 200 nm; (**D**–**H**), 250 nm.

In the molecular layer, immunoreactivity for KChIP3 was found both along the extrasynaptic plasma membrane of dendritic shafts (Figure 3A) and dendritic spines (Figure 3B,C) of PCs, as well as at intracellular sites in those compartments. In addition, KChIP3 immunoparticles were also observed presynaptically outside the active zone of parallel fibre axon terminals (Figure 3B–D). On the other hand, immunoparticles for KChIP3 in the granule cell layer were detected in the somatic plasma membrane of GCs (Figure 3E) and the plasma membrane of GC dendrites, which were distributed in cerebellar glomeruli (Figure 3F–H). KChIP3 immunoparticles were also observed at intracellular sites associated with intracellular membranes in dendrites of GCs and only very few at intracellular sites in somata (Figure 3E–H). Presynaptically, immunoparticles for KChIP3 were detected in mossy fibre terminals, localised along their plasma membrane, but more frequently at intracellular sites (Figure 3F,G). No significant immunolabelling for KChIP3 was found to be associated with inhibitory synapses along the surface of PCs or in Golgi cell axon terminals in cerebellar glomeruli.

**Figure 4:**
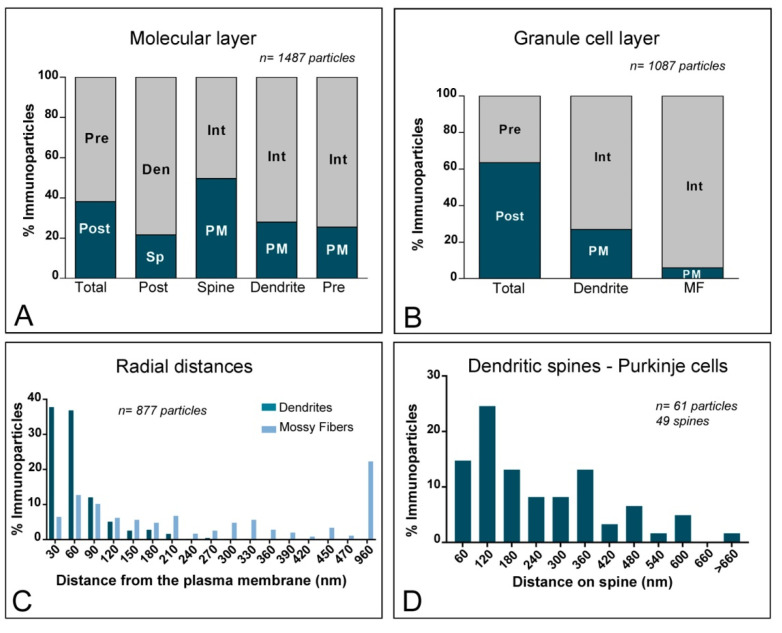
Compartmentalisation of Kv channel interacting protein (KChIP) subunit KChIP3 in cerebellar cells. (**A**) Bar graphs showing the percentage of immunoparticles for KChIP3 at post- and pre-synaptic compartments in the molecular layer. A total of 1487 immunoparticles in the molecular layer were analysed, of which 38.1% were postsynaptic and 61.9% were presynaptic. Postsynaptically, immunoparticles were detected in dendritic spines (21.7%) and in dendritic shafts (78.35%), distributed along the plasma membrane (49.6% in spines; 27.9% in dendrites) and at cytoplasmic sites (50.4% in spines; 72.1% in dendrites). Presynaptically, immunoparticles were detected in parallel fibres of ML (61.9%) distributed along the plasma membrane (25.5%) and at cytoplasmatic sites (74.5%). (**B**) Bar graphs showing the percentage of KChIP3 immunoparticles at post- and pre-synaptic compartments, and along the plasma membrane and intracellular sites in dendritic shafts of granule cells and mossy fibres in the granule cell layer. A total of 1087 immunoparticles in the granular cell layer were analysed, of which 63.5% were postsynaptic and 36.5% were presynaptic. Postsynaptically, immunoparticles were detected in dendrites of GCs (63.5%), distributed along the plasma membrane (27%) and at cytoplasmic sites (73%). Presynaptically, immunoparticles were detected in mossy fibre terminals (36.5%), distributed mostly at cytoplasmic sites (94%) and very few along the plasma membrane (6%). (**C**) Histogram showing the radial distribution of KChIP3 immunoparticles from the plasma membrane towards cytoplasmic sites in dendrites of GCs and mossy fibres. In dendrites, immunoparticles for KChIP3 showed a skewed frequency distribution in the plasma membrane direction, but in mossy fibres they were more equally distributed across the axoplasm. (**D**) Histogram showing the distribution of immunoreactive KChIP3 in relation to glutamate release sites in dendritic spines PCs. The data show the proportion of KChIP3 immunoparticles at a given distance from the edge of the postsynaptic density. About 68.9% of immunolabelled KChIP3 are located in a 60–300 nm wide band, and then the density decreased markedly further in the spine membrane.

To quantitatively assess the localisation of KChIP3 in the cerebellar cortex, we first calculated the proportion of immunoparticles in the plasma membrane and at intracellular sites (Figure 4). From 1487 immunogold particles analysed in the molecular layer, 567 (38.1%) were distributed at postsynaptic sites and 920 (61.9%) at presynaptic sites (Figure 4A). Postsynaptically, 78,3% of all KChIP3 immunoparticles were in dendritic shafts and, of those, 27.9% were located on the plasma membrane and 72.1% at intracellular sites (Figure 4A). In contrast, 21.7% of all KChIP3 immunoparticles were in dendritic spines, with 49.6% of these particles being located on the plasma membrane and 50.4% at intracellular sites (Figure 4A). Presynaptically, from 920 immunogold particles (61.9%) distributed in parallel fibres, 25.5% were located on the plasma membrane and 74.5% at intracellular sites (Figure 4A).

In the granule cell layer, 1087 immunogold particles were analysed and, of those, 690 (63.5%) were distributed at postsynaptic sites and 397 (36.5%) at presynaptic sites (Figure 4B). At postsynaptic sites, 63.5% of KChIP3 immunoparticles were distributed in dendrites of GCs (27% were located on the plasma membrane and 73% at intracellular sites) (Figure 4B). At presynaptic sites, KChIP3 immunoparticles were distributed along the plasma membrane (6% of all presynaptic immunoparticles) and intracellularly (94%) in mossy fibre terminals (Figure 4B).

Next, given the cytoplasmic location of KChIP proteins, from 877 immunogold particles for KChIP3 observed intracellularly, we analysed their radial distribution across dendrites of GCs and mossy fibres (Figure 4C). In GC dendrites, immunoparticles for KChIP3 showed a skewed frequency distribution in the plasma membrane direction. Thus, 74.7% of all particles were located in the first 60 nm beneath the plasma membrane (Figure 4C). In contrast, KChIP3 immunoparticles at mossy fibres were more equally distributed across the axoplasm of mossy fibres, with no preferential association just beneath the presynaptic plasma membrane (Figure 4C).

Then, we investigated the distribution of immunoparticles for KChIP3 in relation to synaptic sites, by measuring the distance from the edge of postsynaptic densities of PC spines to the centre of each gold particle (*n* = 61 immunoparticles on 49 spines) (Figure 4D). The data showed that about 69% of immunoparticles for KChIP3 were distributed within the first 300 nm from the postsynaptic densities, with a reduction in their frequency at further distances in membrane of dendritic spines (Figure 4D).

### 2.3. Subcellular Localisation of KChIP4 in the Cerebellum

**Figure 5:**
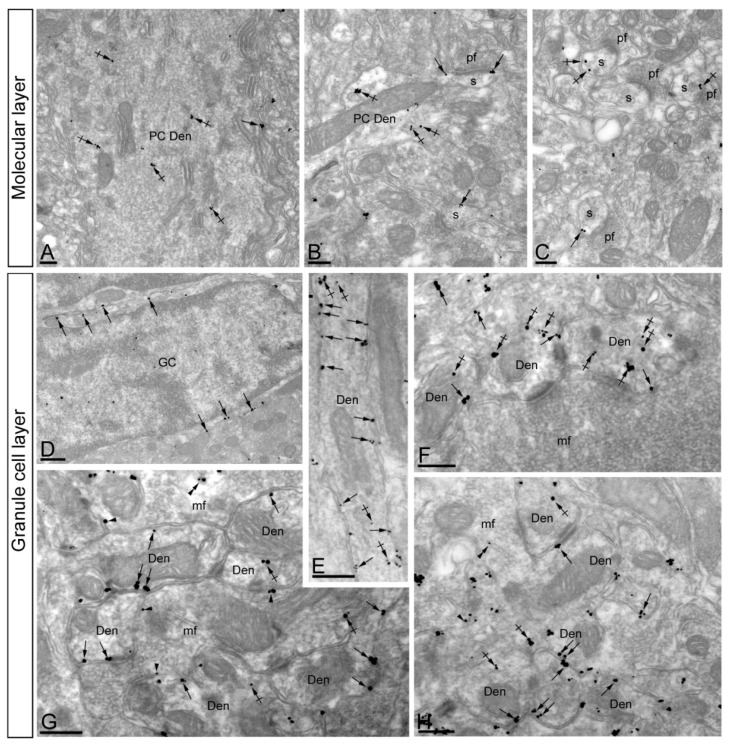
Subcellular localisation of Kv channel interacting protein (KChIP) subunit KChIP4 in the cerebellum. Electron micrographs showing immunoparticles for KChIP4 in the cerebellar cortex, as detected using the pre-embedding immunogold technique. (**A**–**C**) In the molecular layer, KChIP4 immunoparticles were detected in Purkinje cells, distributed along the extrasynaptic plasma membrane (arrows) of dendritic shafts (Den) and dendritic spines (s), as well as at intracellular sites (crossed arrows). (**D**–**H**) In the granule cell layer, immunoparticles for KChIP4 were mostly distributed along the somatic plasma membrane (arrows) and dendrites (Den) of granule cells (GC), as well as at intracellular sites (crossed arrows). At presynaptic sites, immunoparticles for KChIP4 were found along the plasma membrane (arrowheads) and intracellular sites (double arrowheads) of mossy fibre axon terminals (mf). Scale bars: (**A**–**C**,**E**–**H**), 250 nm; (**D**), 500 nm.

**Figure 6:**
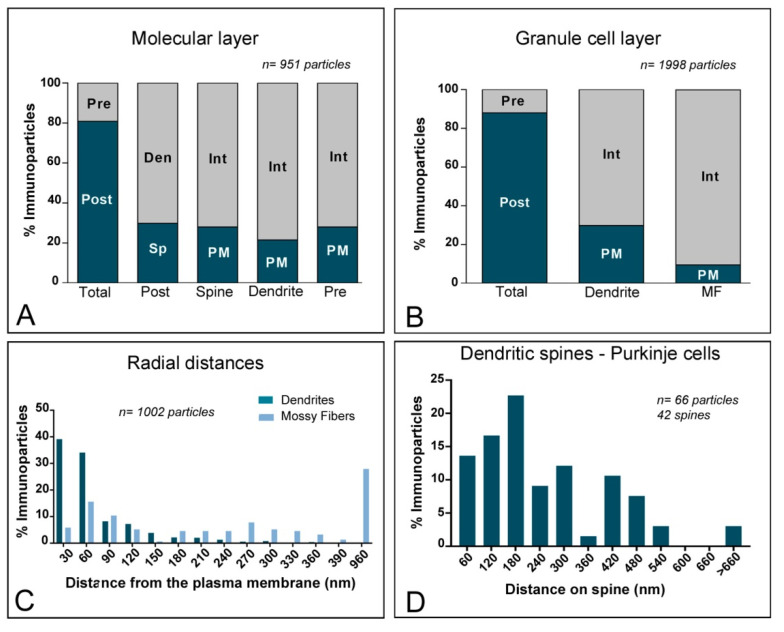
Compartmentalisation of Kv channel interacting protein (KChIP) subunit KChIP4 in cerebellar cells. (**A**) Bar graphs showing the percentage of immunoparticles for KChIP4 at post- and pre-synaptic compartments in the molecular layer. A total of 951 immunoparticles in the molecular layer were analysed, of which 80.9% were postsynaptic and 19.1% were presynaptic. Postsynaptically, immunoparticles were detected in dendritic spines (29.8%) and in dendritic shafts (70.2%), distributed along the plasma membrane (27.9% in spines; 21.5% in dendrites) and at cytoplasmic sites (72.1% in spines; 78.5% in dendrites). (**B**) Bar graphs showing the percentage of KChIP4 immunoparticles at post- and pre-synaptic compartments, and along the plasma membrane and intracellular sites in dendritic shafts of granule cells and mossy fibres in the granule cell layer. A total of 1998 immunoparticles in the granular cell layer were analysed, of which 87.9% were postsynaptic and 12.1% were presynaptic. Postsynaptically, immunoparticles were detected in dendrites of GCs (87.9%), distributed along the plasma membrane (29.9%) and at cytoplasmic sites (70.1%). Presynaptically, immunoparticles were detected in mossy fibre terminals (12.1%), distributed mostly at cytoplasmic sites (90.4%) and very few along the plasma membrane (9.6%). (**C**) Histogram showing the radial distribution of KChIP4 immunoparticles from the plasma membrane towards cytoplasmic sites in dendrites of GCs and mossy fibres. In dendrites, immunoparticles for KChIP4 showed a skewed frequency distribution in the plasma membrane direction, but in mossy fibres, they were more equally distributed across the axoplasm. (**D**) Histogram showing the distribution of immunoreactive KChIP4 in relation to glutamate release sites in dendritic spines PCs. The data show the proportion of KChIP4 immunoparticles at a given distance from the edge of the postsynaptic density. About 74.2% of immunolabelled KChIP4 are located in a 60–300 nm wide band, and then the density decreased markedly further in the spine membrane.

Similar to the distribution of KChIP3, immunoreactivity for KChIP4 in the molecular layer was observed in the extrasynaptic plasma membrane of dendritic shafts and dendritic spines of PCs, and associated with membranes at intracellular sites (Figure 5A–C). Immunoparticles for KChIP4 were also detected at presynaptic sites in the extrasynaptic plasma membrane of parallel fibre axon terminals, but less frequently than immunoparticles for KChIP3. Quantitatively, from 951 immunogold particles analysed in the molecular layer, 769 (80.9%) were distributed at postsynaptic sites and 131 (19.1%) at presynaptic sites (Figure 6A). Postsynaptically, 29.8% of all KChIP4 immunoparticles were in dendritic spines and, of those, 27.9% were located on the plasma membrane and 72.1% at intracellular sites (Figure 6A). In contrast, 70.2% of all KChIP4 immunoparticles were in dendritic shafts, with 21.5% of these particles being located on the plasma membrane and 78.5% at intracellular sites (Figure 6A). Presynaptically, from 131 immunogold particles (19.1%) distributed in parallel fibres, 28% were located on the plasma membrane and 72% at intracellular sites (Figure 6A).

In the granule cell layer, KChIP4 immunoparticles were distributed in plasma membrane of GC somata (Figure 5D) and their dendrites located in the cerebellar glomeruli (Figure 5E–H), as well as at intracellular sites associated with cytoplasmic membranes in dendrites of GCs (Figure 5E–H). At presynaptic sites, KChIP4 immunoparticles were distributed along the plasma membrane and intracellularly in mossy fibre terminals (Figure 5F–H). This was confirmed using quantitative analyses showing that from 1998 immunogold particles analysed in the granule cell layer, 1757 (87.9%) were distributed postsynaptically and 241 (12.1%) presynaptically (Figure 6B). Postsynaptically, 87.9% of immunoparticles were detected in dendritic shafts of granule cells, with 29.9% located on the plasma membrane and 70.1% at intracellular sites (Figure 6B). In contrast, in mossy fibres, many immunoparticles were distributed intracellularly (90.4% of all presynaptic immunoparticles) and few (9.6%) along the plasma membrane (Figure 6B). No significant immunolabelling for KChIP4 was found to be associated with inhibitory synapses along the surface of PCs or in Golgi cell axon terminals in cerebellar glomeruli.

Next, we analysed the radial distribution of intracellular immunoparticles for KChIP4 across dendrites of GCs and mossy fibres (Figure 6C). Similar to KChIP3, 73.2% of all immunoparticles for KChIP4 were located in the first 60 nm beneath the plasma membrane, thus showing a skewed frequency distribution towards the plasma membrane direction (Figure 6C). At mossy fibres, in contrast, KChIP4 immunoparticles showed no preferential association just beneath the presynaptic plasma membrane, but instead a more equal distribution across the axoplasm (Figure 6C). Overall, our data suggest that both KChIP3 and KChIP4 have the same radial distribution.

To determine the location of KChIP4 immunoparticles relative to glutamate release sites, we analysed the position of immunoparticles in relation to the closest edge of the postsynaptic densities (PSDs) of PC spines (Figure 6D). This analysis showed that approximately 74.2% of immunoparticles (*n* = 66 immunoparticles on 42 spines) were located within a distance of 300 nm from the edge of the PSDs (Figure 6D).

### 2.4. Subcellular Localisation of Kv4 in the Cerebellum

**Figure 7:**
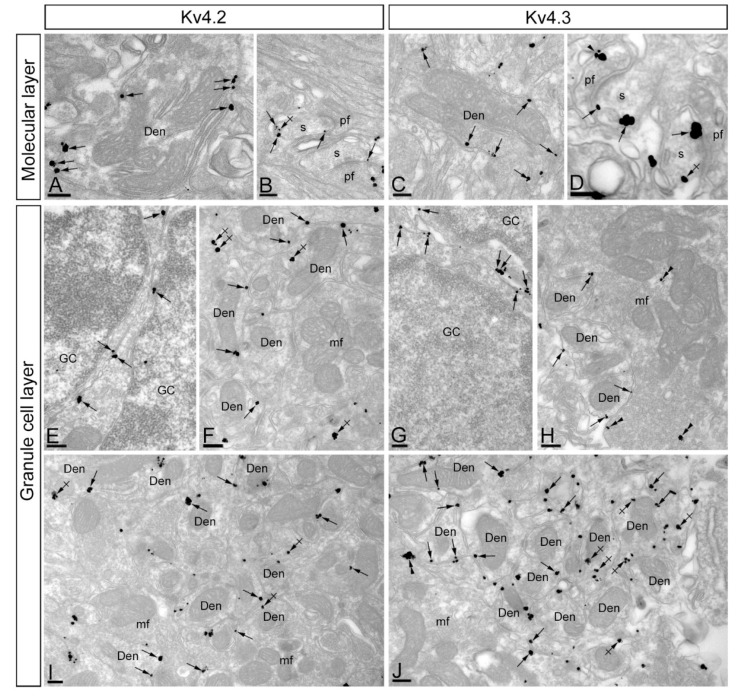
Subcellular distribution of voltage-gated potassium (Kv) channel subunits Kv4.2 and Kv4.3 channels. Immunoreactivity for Kv4.2 and Kv4.3 in the cerebellar cortex as demonstrated by pre-embedding immunogold labelling. (**A**–**D**) Immunoparticles for both Kv4.2 and Kv4.3 in the molecular layer were found in the plasma membrane (arrows) and intracellular sites (crossed arrows) of PC dendrites (Den) and spines (s) establishing synapses with parallel fibres (pf). Although, at low frequency, Kv4.3 immunoparticles were also found in the plasma membrane (arrowheads) of parallel fibres (pf). (**E**–**J**) Immunoparticles for both Kv4.2 and Kv4.3 in the granule cell layer were found in the plasma membrane (arrows) of GC somata and GC dendrites (Den) in cerebellar glomeruli. In addition, immunolabelling was found intracellularly (crossed arrows). Few immunoparticles were also observed presynaptically along the plasma membrane (arrowheads) and intracellular sites (double arrowheads) in mossy fibre (mf) axon terminals. Scale bars: (**A**–**J**), 200 nm.

**Figure 8:**
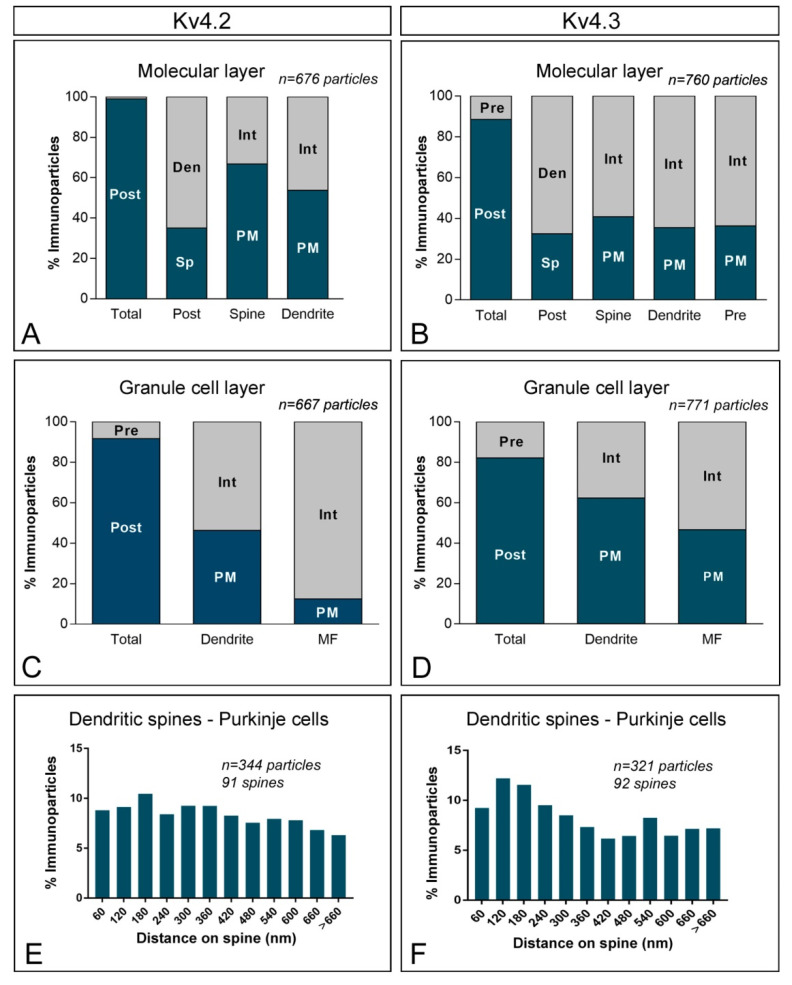
Compartmentalisation of voltage-gated potassium (Kv) channel subunits Kv4.2 and Kv4.3 in cerebellar cells. (**A**,**B**) Bar graphs showing the percentage of immunoparticles for Kv4.2 and Kv4.3 in neuronal compartment in the molecular layer. Immunoparticles for Kv4.2 were mostly localised at the postsynaptic compartment (99% of all particles), while Kv4.3 was distributed at postsynaptic (88.4%) and presynaptic (11.6%) compartments. (**C**,**D**) Bar graphs showing the percentage of immunoparticles for Kv4.2 and Kv4.3 in the neuronal compartment in the granule cell layer. A total of 667 immunoparticles for Kv4.2 and 771 for Kv4.3 were analysed. Postsynaptically, immunoparticles were detected in dendrites of GCs (91.6% for Kv4.2; 82.2% for Kv4.3), distributed along the plasma membrane (46.3% for Kv4.2; 62.3% for Kv4.3) and at cytoplasmic sites (53.7% for Kv4.2; 37.7% for Kv4.3). Presynaptically, immunoparticles were detected in mossy fibre terminals (8.4% for Kv4.2; 17.8% for Kv4.3), distributed at cytoplasmic sites (87.5% for Kv4.2; 53.3% for Kv4.3) and along the plasma membrane (12.5% for Kv4.2; 46.7% for Kv4.3). (**E**,**F**) Histogram showing the distribution of immunoparticles for Kv4.2 and Kv4.3 in relation to glutamate release sites in dendritic spines of PCs. About 46% of immunolabelled Kv4.2 and 52% of immunolabelled Kv4.3 were located in a 60–300 nm wide band. These data show that Kv4.2 immunoparticles were more equally distributed along PC spines, while immunoparticles Kv4.3 were skewed toward the PSD of PC spines.

To investigate the subcellular localisation of Kv4.2 and Kv4.3 in the cerebellar cortex, we used the same pre-embedding immunogold approach and type of quantitative analyses as previously for KChIP3 and KChIP4. Overall, Kv4.2 and Kv4.3 exhibit similar ultrastructural localisation (Figure 7A–J), with clear labelling of somatic and dendritic membranes. In the molecular layer (Figure 7A–D), immunoreactivity for Kv4.2 and Kv4.3 was distributed in plasma membrane of dendritic spines and shafts of PCs (57.7% of Kv4.2; 37.2% of Kv4.3) and also associated at intracellular sites with cytoplasmic membranes in their dendritic shafts and spines (42.3% of Kv4.2; 62.8% of Kv4.3) (Figure 7A–D and Figure 8A,B). Immunoparticles for Kv4.2 and Kv4.3 also appeared occasionally at the edge of asymmetrical synapses on dendritic spines (Figure 7B,D). Presynaptically, there is a clear difference between Kv4.2 and Kv4.3 (Figure 7A–D and Figure 8A,B). Thus, while immunoreactivity for Kv4.2 were occasionally detected in parallel fibres establishing asymmetrical synapses with dendritic spines of PCs (1% of all particles), immunoreactivity for Kv4.3 were more frequently observed (11.6% of all particles) in those compartments (Figure 7A–D and Figure 8A,B). Immunoparticles for Kv4.3 were mainly localised along the extrasynaptic plasma membrane of parallel fibres (Figure 7D).

In the granule cell layer, immunoparticles for Kv4.2 (Figure 7E,F,I) and Kv4.3 (Figure 7G,H,J) were primarily observed in the somatic plasma membrane of GCs (Figure 7E,G) and their dendrites (Figure 7F,H–J) located in cerebellar glomeruli (46.3% of Kv4.2 immunoparticles; 62.3% of Kv4.3 immunoparticles). Kv4.2 and Kv4.3 immunoparticles were also found at intracellular sites in dendrites of GCs (53.7% of Kv4.2 immunoparticles; 37.7 of Kv4.3 immunoparticles) (Figure 8C,D). Presynaptically, immunoparticles for Kv4.2 and Kv4.3 in mossy fibres were distributed along the plasma membrane (12.5% for Kv4.2 and 46.7% for Kv4.3), but more frequently at intracellular sites (87.5% for Kv4.2 and 53.3% for Kv4.3) (Figure 7F,H–J and Figure 8C,D). Thus, the localisation of Kv4.2 and Kv4.3 channels is associated with excitatory synapses formed between GC dendrites and mossy fibres. No significant immunolabelling for Kv4.2 or Kv4.3 was found to be associated with inhibitory synapses along the surface of PCs or in Golgi cell axon terminals in cerebellar glomeruli. Overall, the subcellular distribution pattern of Kv4.2 and Kv4.3 channels shown here is very similar to that described above for KChIP3 and KChIP4.

Finally, we investigated the location of Kv4.2 and Kv4.3 immunoparticles relative to glutamate release sites. Analysing the position of immunoparticles in relation to the closest edge of the PSD of PC spines (*n* = 344 immunoparticles on 91 spines for Kv4.2; *n* = 321 immunoparticles on 92 spines for Kv4.3), we found that 46% of immunoparticles for Kv4.2 and 52% of immunoparticles for Kv4.3 were located within a distance of 300 nm from the edge of postsynaptic densities (PSDs) (Figure 8E,F). In addition, immunoparticles Kv4.2 were more equally distributed along PC spines, while immunoparticles Kv4.3 were skewed toward the PSD of PC spines (Figure 8E,F). This data suggests that Kv4.2 and Kv4.3 channels showed a differential distribution in PC spines.

## 3. Discussion

Many neuron populations express a voltage-dependent K^+^ current with rapid activation and inactivation, known as A-type K^+^ currents [7,8]. Somato-dendritic A-type Kv channels are formed from Kv4 α subunits in molecular and functional partnership with KChIP subunits [11,20]. However, the association of KChIP subunits with Kv4 channels is different depending on the brain region and the neuron population [20,22]. Although the presence of A-type K^+^ currents has been reported in cerebellar neurons [25,26], the molecular characterisation of the channels and associated proteins responsible for such currents has not yet been fully provided. Here, we investigated the subcellular localisation patterns of KChIP with Kv4 subunits and unravelled the overlapping subcellular localisation of KChIP3, KChIP4, Kv4.2 and Kv4.3 in the same compartments of PCs and GCs, and their presynaptic localisation in different synaptic inputs. Our data suggest complex requirements for precise compositions of Kv4 α subunits and KChIPs in channel complexes.

### 3.1. KChIP3 and KChIP4 Are Expressed in Purkinje Cells and Granule Cells

Previous in situ hybridisation studies demonstrated that the four KChIP subunits are widely expressed throughout the cerebellum [19]. Consistent with these earlier observations, we found KChIP3 and KChIP4 expression across several cerebellar neuron populations, showing overlapping patterns and remarkable correspondence with the cellular expression of Kv4.2 and Kv4.3. Acting as auxiliary subunits, the main role of KChIP subunits is to facilitate Kv4 assembly, and to modulate their surface density and electrical properties [11,20,27]. To accomplish those roles efficiently, all four KChIPs and Kv4 α subunits are molecularly associated in native brain, as demonstrated using co-immunoprecipitation techniques [20,21]. KChIPs are widely expressed throughout the brain, and whereas in many regions their cellular expression patterns are complementary, there are also neuron populations in which they are co-expressed [19]. It seems now clear that the association KChIP subunits with Kv4 channels and their patterns of distribution are different depending on the brain region and the neuron population [20,22]. For example, KChIP2, KChIP3 and KChIP4 were shown to colocalise with Kv4.2 in excitatory neurons in hippocampal CA1 and cortical pyramidal cells, while KChIP1 colocalise with Kv4.3 in inhibitory interneurons in the hippocampus, cerebral cortex and striatum [20]. Similarly, all four KChIP and all three Kv4 α subunits are expressed in the basal lateral amygdala, but only KChIP1 and Kv4.3 are preferentially expressed in interneurons [21].

The cerebellum exemplifies a brain region where differential co-distribution pattern between individual KChIPs with Kv4.2 or Kv4.3 is not taking place. Thus, our data show that KChIP3 and KChIP4 were expressed in the same neuronal populations as Kv4.2 and Kv4.3, and this included PCs and GCs. The present findings are in agreement with previous studies showing that the mRNA and protein for KChIP3 and KChIP4 are distributed in PCs and GCs [19,22], two neuron populations that also express Kv4.2 and Kv4.3 transcripts and proteins [22,23,28,29,30]. Interestingly, our single-label immunoperoxidase immunohistochemistry also showed that only KChIP3 and Kv4.3 were found in interneurons (basket and stellate cells) in the molecular layer, suggesting that some cerebellar neurons also show differential association between KChIPs and Kv4. However, the specificity of interaction between individual KChIPs and a particular type of Kv4 channel in the cerebellum remains to be investigated biochemically. Altogether, the co-distribution between KChIPs with Kv4.2 or Kv4.3 in PCs and GCs, and the observation of differential distribution in cerebellar interneurons, supports previous reports on interaction of KChIPs with Kv4 channels in vivo. Although the light microscopy immunohistochemical evidence is highly suggestive, definitive proof that KChIP3, KChIP4, Kv4.2 and Kv4.3 are distributed in the same subcellular compartment requires electron microscopy, as discussed next.

### 3.2. KChIP and Kv4 Compartmentalisation in Cerebellar Neurons

The effect of Kv4 channels on a given neuron directly depends on its electrical properties and the neuronal compartment where they are inserted into the plasma membrane [31], and additional mechanisms also involve their association with KChIP proteins [7,8]. Characterising the distribution and abundance of KChIP proteins and their spatial relation with Kv4 channels is essential to understand how information is processed between the different neurons in the cerebellum. To this end, the present study provides the first systematic and comprehensive quantitative description on the subcellular localisation of the KChIP and Kv4 subunits along the neuronal surface of cerebellar neurons. Thus, using high-resolution immunoelectron microscopy, we unravelled previously uncharacterised KChIP3, KChIP4, Kv4.2 and Kv4.3 subcellular localisation in PCs and GCs.

PCs are the main output neurons of the cerebellar cortex and their dendritic arborisations lie in the molecular layer, which also contains interneurons that relay information to PCs [4]. Our immunoelectron microscopy study revealed very similar subcellular distribution of KChIP3, KChIP4, Kv4.2 and Kv4.3 throughout the dendritic compartments of PCs. In the molecular layer, the signal for the two KChIP subunits and the two Kv4 α subunits was observed primarily in both the dendrites and spines of PCs. The quantitative comparison of PC spines revealed that around 70% of all immunoparticles for KChIP3 and KChIP4 and around half of all immunoparticles for Kv4.2 and Kv4.3 were located within a distance of 300 nm from the edge of postsynaptic specialisation. Given that PC spines are the primary point-of-contact parallel fibres, the axon terminals of granule cells [4], the close relation of KChIP and Kv4 α subunits with the postsynaptic specialisation suggest that Kv4.2 and Kv4.3 channel activity could directly regulate excitatory synaptic transmission in PCs. Consistent with this idea, deletion of Kv4.2 resulted in alteration of specific forms of synaptic plasticity in the hippocampus [32], and mutations affecting Kv4.3 and KChIP4 in PCs cause cerebellar ataxias [33,34].

GCs are located strategically within the cerebellar circuitry to play a role as a main input source of cerebellum. Previous electrophysiological studies demonstrated that GCs generate a high density of A-type current reflecting the expression of Kv4 channels [23,35]. In agreement with our light microscopy immunohistochemistry, results from our immunoelectron microscopy study revealed strikingly similar subcellular distribution of KChIP3 and KChIP4, as well as Kv4.2 and Kv4.3, throughout the somato-dendritic compartments of GCs. Similar somato-dendritic distributions of Kv4 have been described previously in other brain regions, including the hippocampus, cortex, parasubiculum and hypothalamus [24,36,37,38,39].

The significance for the expression of multiple KChIP subunits in the same neuronal population is not clear. Multiple KChIP expressions may allow region-specific targeting of Kv4 channels to different cellular compartments [7]. This does not seem to be the case, because one of the main findings from our study is that both KChIP3 and KChIP4 share the same subcellular localisation in the same neuronal compartments. Additionally, different KChIP isoforms can alter the electrical properties of Kv4 channels [40]. We could not evaluate this possibility, because our antibodies do not distinguish between different KChIP splice variants. Nevertheless, the similar somato-dendritic distribution found for KChIP3, KChIP4, Kv4.2 and Kv4.3 along the neuronal surface of PCs and GCs favours the idea that the four proteins are part of macromolecular complexes likely forming heterotetrameric Kv4.2/Kv4.3 channels. The Kv4 channel is composed of four α subunits, which can form heteromeric or homomeric channels [7], with a stoichiometry KChIP-Kv4 4:4 that is variable in composition [41]. Therefore, it is possible that KChIP3 and/or KChIP4 may regulate the activity of A-type channels containing Kv4.2 and Kv4.3 subunits in PCs and GCs. This is consistent with co-immunoprecipitation experiments reporting co-association between Kv4.2 and Kv4.3 [20] that may help to explain the co-existence of the four proteins in the same neuronal type. In this scenario, we cannot discount the role of KChIP1 binding to different Kv4 [22,42].

### 3.3. Other Subcellular Locations for KChIP and Kv4 α Subunits Are Possible

The strong membrane-associated immunolabelling for KChIP3 and KChIP4 in GC somata and dendrites likely reflect their association with the transmembrane Kv4.2 and Kv4.3. However, a large number of immunoparticles (40% for KChIP3 and 40% KChIP4) were observed at intracellular sites, consistent with studies reporting a diffuse cytoplasmic localisation when expressed in heterologous cells and primary cultures [14]. One possibility is that this could represent pools of KChIP3 and KChIP4 binding to presenilin [43], meaning that they could have a function different to that of Kv4 channel auxiliary subunits. A more likely explanation is that such cytoplasmic distribution could represent an excess of KChIP3 and KChIP4 that might co-assemble with Kv4.2 and/or Kv4.3. The data presented here showing that 70% of all cytoplasmic-associated immunoparticles are very close to the plasma membrane, just at a short distance of 60 nm, support this idea. This suggests that the interaction between KChIPs and Kv4 channels might be established before they transit to the neuronal surface, as has been demonstrated previously for other ion channels [44].

Unexpectedly, in addition to the described distribution in somato-dendritic compartments of PCs and GCs, we also found all KChIP and Kv4 subunits in presynaptic axon terminals, but showing differential distribution and abundance depending on the synaptic input. Thus, parallel fibres contained a large proportion of presynaptic KChIP3 (62% of immunoparticles), moderate proportion of KChIP4 (19% of immunoparticles), small amount of Kv4.3 (12% of immunoparticles) and absence of Kv4.2 (1% of immunoparticles). However, KChIP3, KChIP4, Kv4.2 and Kv4.3 (36%, 12%, 8% and 18% of all immunoparticles, respectively) were detected in mossy fibre terminals. Because there are few published high-resolution immunoelectron microscopic and electrophysiological data showing the precise localisation of KChIP and Kv4 subunits in the cerebellum, it is difficult to draw conclusions about the functional significance of our results at present. On the basis of previous and present results, one possibility is that Kv4 and KChIP are related with LTP at the mossy fibre–granule cell, known to be regulated by Kv4 channels [30]. Given that this LTP has also been attributed to presynaptic mechanisms [45], further electrophysiological studies will be essential to unravel a specific role carried out by KChIP and Kv4 α subunits channels located at axon terminals.

## 4. Material and Methods

### 4.1. Animals

We used four adult male Wistar rats (weight: 200–250 g) obtained from Charles River Laboratories (Barcelona, Spain) and kept in the Animal House Facilities of the Universidad de Castilla-La Mancha (Albacete, Spain). At any step of experimental procedures, care and handling of animals were in accordance with Spanish (RD 1201/2015) and European Union regulations (86/609/EC), and all protocols and methodologies were approved by the local Animal Care and Use Committee (PR-2014-07-05, 19 July 2014).

Animals were deeply anesthetised by intraperitoneal injection of ketamine-xylazine 1:1 (0.1 mL/kg, body weight). Once reflex activity was completely abolished, the heart was surgically exposed for perfusion fixation through the ascending aorta, first with 0.9% saline and then followed by 400 mL of freshly prepared ice-cold fixative containing 4% paraformaldehyde, 0.05% glutaraldehyde and ~0.2% picric acid, made up in 0.1 M phosphate buffer (PB; pH 7.4). After perfusion, brains were removed and immersed in the same fixative for 2 h or overnight at 4 °C. Tissue blocks were washed thoroughly in 0.1 M PB. Coronal 60 μm thick sections were cut on a Vibratome (Leica V1000).

### 4.2. Antibodies and Chemicals

We used the following primary antibodies from NeuroMab (UC Davis/NIH, Davis, CA, USA): (1) monoclonal anti-Kv4.2 (K57/1; amino acid. 209–225 of human Kv4.2, Q9NZV8), (2) monoclonal anti-Kv4.3 (K75/41; aa. 415–636 of rat Kv4.3, Q62897), (3) monoclonal anti-KChIP3 (K665/38; amino acid 1–256 of rat KChIP3, Q9JM47) and (4) monoclonal anti-KChIP4 (K55/7; amino acid 1–233 of rat KChIP3, Q3YAB7). The characteristics and specificity of these antibodies using the corresponding knockout mice have been described by the manufacturer (http://neuromab.ucdavis.edu/catalog.cfm, UC Davis/NIH, Davis, CA, USA). Other primary antibodies used were the following: (1) rabbit anti-Kv4.2 polyclonal (APC-023; Alomone Labs., Jerusalem, Israel), (2) rabbit anti-Kv4.3 polyclonal (APC-017; Alomone Labs., Israel) and (3) rabbit anti-KChIP3 polyclonal (Rb-Af400; aa. 239–256 of rat KChIP3, MM_032462; Frontier Institute Co., Sapporo, Japan) antibodies.

We used the following secondary antibodies: (1) biotinylated goat anti-mouse IgG and biotinylated goat anti- guinea pig IgG (Vector Laboratories, Burlingame, CA, USA) and (2) goat anti-guinea pig and anti-mouse IgG coupled to 1.4 nm gold (1:100; Nanoprobes Inc., Stony Brook, NY, USA).

### 4.3. Immunohistochemistry for Light Microscopy

Immunohistochemical reactions at the light microscopic level were carried out using the immunoperoxidase method as described earlier [46]. Briefly, sections were incubated in 10% normal goat serum (NGS) diluted in 50 mMTris buffer (pH 7.4) containing 0.9% NaCl (TBS), with 0.2% Triton X-100, for 1 h. Sections were incubated in anti-KChIP3, anti-KChIP4, anti-Kv4.2 or anti-Kv4.3 (1–2 µg/mL diluted in Tris-buffered saline TBS containing 1% NGS), followed by incubation in biotinylated goat anti-rabbit IgG or biotinylated goat anti-mouse IgG (Vector Laboratories, Burlingame, CA, USA) diluted 1:200 in TBS containing 1% NGS. Sections were then transferred into avidin–biotin–peroxidase complex (ABC kit, Vector Laboratories). Bound peroxidase enzyme activity was revealed using 3,3’-diaminobenzidine tetrahydrochloride (DAB; 0.05% in TB, pH 7.4) as the chromogen and 0.01% H_2_O_2_ as the substrate. Finally, sections were air-dried and mounted prior to observation in a Leica photomicroscope (DM2000) equipped with differential interference contrast optics and a digital imaging camera.

### 4.4. Immunohistochemistry for Electron Microscopy

Immunohistochemical reactions for electron microscopy were carried out using the pre-embedding immunogold method described previously [46]. Briefly, free-floating sections were incubated in 10% (*v*/*v*) NGS diluted in TBS. Sections were then incubated in anti-KChIP3, anti-KChIP4, anti-Kv4.2 or anti-Kv4.3 (3–5 μg/mL diluted in TBS containing 1% (*v*/*v*) NGS), followed by incubation in goat anti-rabbit IgG or anti-mouse IgG coupled to 1.4 nm gold (Nanoprobes Inc., Stony Brook, NY, USA), respectively. Sections were post-fixed in 1% (*v*/*v*) glutaraldehyde and washed in double-distilled water, followed by silver enhancement of the gold particles with an HQ Silver kit (Nanoprobes Inc., Stony Brook, NY, USA). Sections were then treated with osmium tetraoxide (1% in 0.1 m phosphate buffer), block-stained with uranyl acetate, dehydrated in graded series of ethanol and flat-embedded on glass slides in Durcupan (EMS, Hatfield, PA, USA) resin. Regions of interest were cut at 70–90 nm on an ultramicrotome (Reichert Ultracut E, Leica, Vienna, Austria) and collected on single slot pioloform-coated copper grids. Staining was performed on drops of 1% aqueous uranyl acetate followed by Reynolds’s lead citrate. Ultrastructural analyses were performed in a Jeol-1010 electron microscope.

### 4.5. Quantification of KChIPs and Kv4 Channel Immunoreactivities

To establish the relative abundance of KChIP3, KChIP4, Kv4.2 and Kv4.3, immunoreactivity in different compartments of cerebellar neurons, we used 60 μm thick coronal slices processed for pre-embedding immunogold immunohistochemistry. The procedure was similar to that used previously [46,47]. Briefly, for each of three adult animals, three samples of tissue were obtained for the preparation of embedding blocks (totalling nine blocks). To minimise false negatives, electron microscopic serial ultrathin sections were cut close to the surface of each block, as immunoreactivity decreased with depth. We estimated the quality of immunolabelling by always selecting areas with optimal gold labelling at approximately the same distance from the cutting surface. Randomly selected areas were then photographed from the selected ultrathin sections and printed with a final magnification of 45,000×. Quantification of immunogold labelling was carried out in reference areas totalling approximately 2500 µm^2^. Quantification of immunolabelling was performed in three different ways:

Percentage of immunoparticles for KChIP and Kv4 subunits: To study the frequency of KChIP3, KChIP4, Kv4.2 and Kv4.3, we counted immunoparticles identified in each reference area and present in different subcellular compartments: dendritic spines, dendritic shafts and axon terminals. The data were expressed as a percentage of immunoparticles in each subcellular compartment, both in the plasma membrane and at intracellular sites.

Radial distribution of KChIP proteins across dendrites and mossy fibres: Given that KChIP proteins are cytoplasmic, from the pool of immunoparticles that were not membrane-associated, we next determined how close KChIP3 (*n* = 877 gold particles) and KChIP4 (*n* = 1002 gold particles) immunoparticles are from the plasma membrane in dendrites of granule cells and mossy fibres in the granule cell layer. The distance from the border between the plasma membrane of the dendrite and the mossy fibre to the centre of each immunoparticle was measured along an axis perpendicular to the plasma membrane. The distances of immunoparticles were sorted into bins of 30 nm.

Distribution of Kv4 and KChIP proteins relative to glutamate release sites: To determine the relative abundance of KChIP3, KChIP4, Kv4.2 and Kv4.3 in dendritic spines of Purkinje cells relative to excitatory synapses with parallel fibres, immunoparticles identified in each reference area were counted. Next, the length of the dendritic spine membrane from the edge of the synaptic junction was measured. Then, the distance between the closest edge of the postsynaptic density and the centre of the immunoparticles was measured along the PC spine membrane (*n* = 61 for KChIP3; *n* = 66 for KChIP4; *n* = 344 for Kv4.2; *n* = 321 for Kv4.3) using a digitising tablet and appropriate software (ImageJ). Finally, to obtain a normalised value of the relative abundance of four proteins along the PC dendritic spines, the number of gold particles was expressed as relative frequency in bins corresponding to 60 nm membrane segments of spine membrane.

All data used and/or analysed during the current study are available from the corresponding author on reasonable request. All co-authors of the present manuscript can certify that it has not been submitted to more than one journal for simultaneous consideration and that the manuscript has not been published previously (partly or in full). The authors also can certify that our main study is not split up into several parts to increase the quantity of submissions, that none of the data presented here have been fabricated or manipulated and that we present our own data/text/theories/ideas. All authors and authorities have explicitly provided their consent to submit the present manuscript and in general, we all agree with the ethical responsibilities of authors of the journal. Finally, all authors give consent for publication in *International Journal of Molecular Sciences*.

### 4.6. Controls

To test method specificity in the procedures for light and for electron microscopy, the primary antibodies were either omitted or replaced with 5% (*v*/*v*) normal serum of the species of the primary antibody (Supplementary Figures S1 and S2). Under these conditions, no selective labelling was observed. In addition, labelling patterns obtained for light and electron microscopy obtained using monoclonal antibodies were compared with labelling obtained using polyclonal antibodies, obtaining the same intensities and patterns of distribution. Finally, labelling patterns were also compared to those obtained by Calbindin (Swant, Marly, Switzerland). Only the antibodies against KChIP3, KChIP4, Kv4.2 and Kv4.3 consistently labelled the plasma membrane.

## Supplementary Materials

Supplementary materials can be found at https://www.mdpi.com/1422-0067/21/17/6403/s1. Supplementary Figure S1: Method specificity in the procedures for light microscopy using an immunoperoxisade method. Supplementary Figure S2: Method specificity in the procedures for electron microscopy using a pre-embedding immunogold method.
