# Supplementary material for: Cellular and Subcellular Localisation of Kv4-Associated KChIP Proteins in the Rat Cerebellum
Source: Int J Mol Sci. 2020 Sep 3;21(17):6403. doi: 10.3390/ijms21176403 (PMC7503578; doi:10.3390/ijms21176403)
Supplement: Supplementary file 1 [file ijms-21-06403-s001.pdf]

<u>SUPPLEMENTARY MATERIAL</u>

Cellular and subcellular localisation of Kv4-associated KChIP proteins in the rat cerebellum

Alfaro-Ruiz et al.

<u>SUPPLEMTARY FIGURE 1</u>

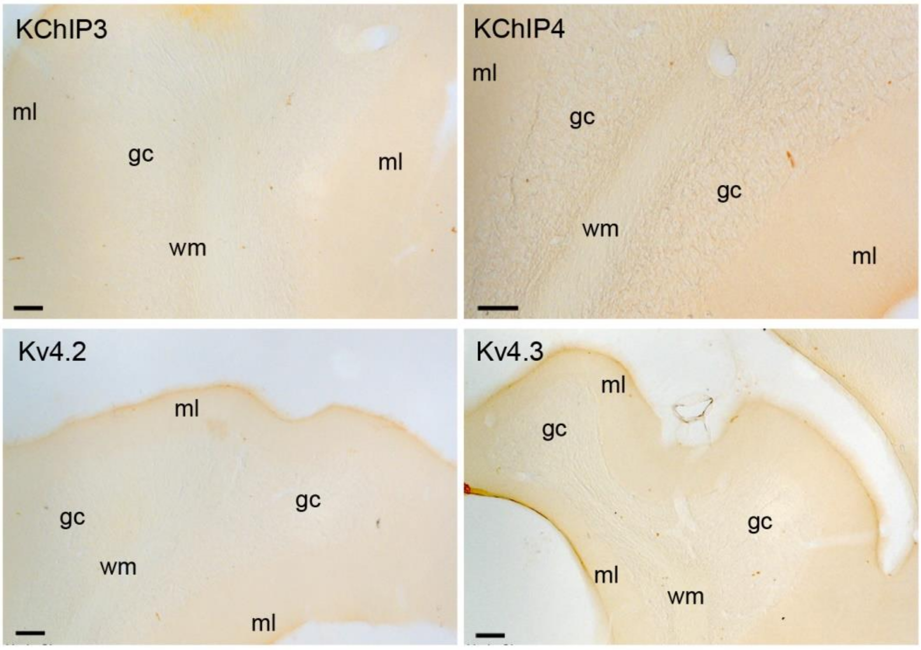

Supplementary Figure 1. Method specificity in the procedures for light microscopy using an immunoperoxisade method. The primary antibodies were replaced with 5% (v/v) normal serum. Under these conditions, immunolabelling for KChIP3 (panel A), KChIP4 (panel B), Kv4.2 (panel C) or Kv4.3 (panel D) was dramatically reduced or mostly absent from the cerebellar cortex. Scale bars: A-D, 30 µm.

SUPPLEMTARY FIGURE 2

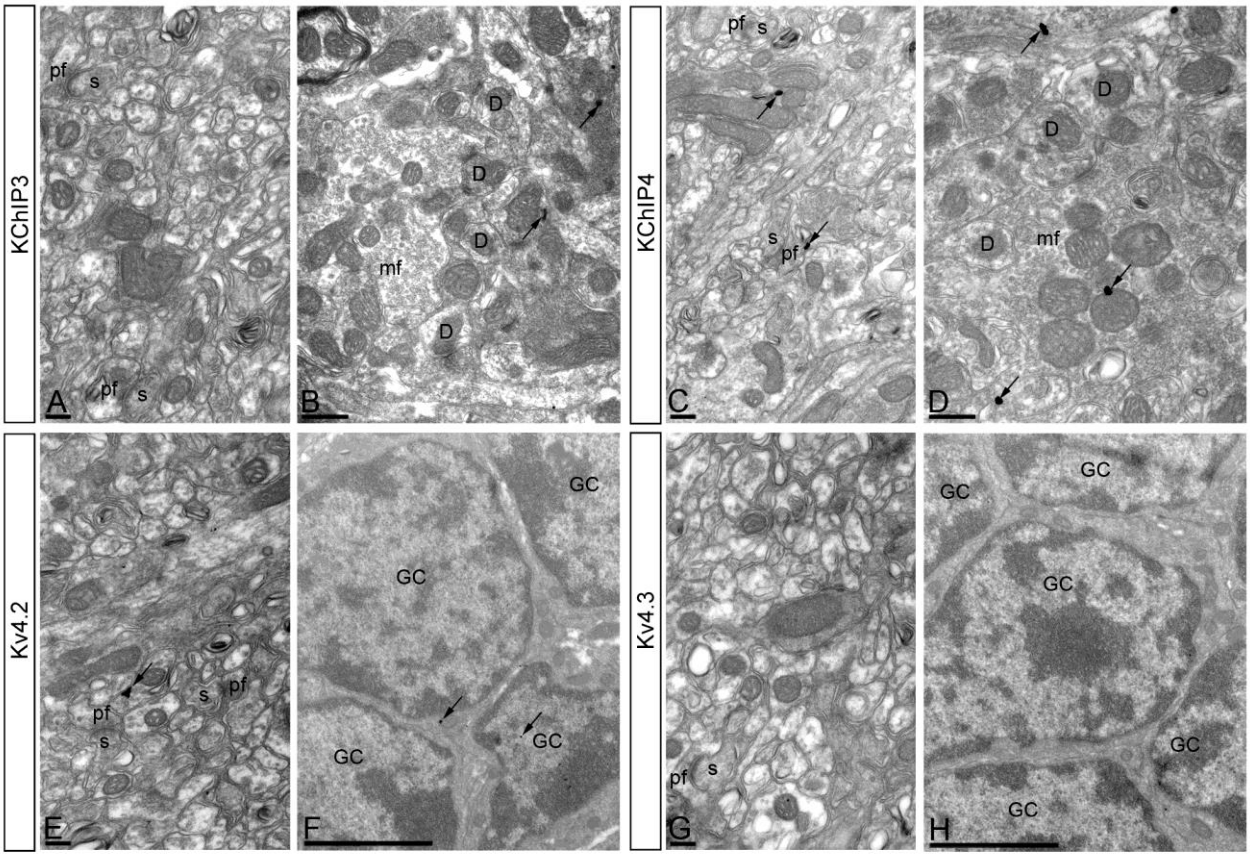

Supplementary Figure 2. Method specificity in the procedures for electron microscopy using a pre-embedding immunogold method. The primary antibodies were replaced with 5% (v/v) normal serum. Under these conditions, immunolabelling for KChIP3 in the molecular layer (panel A) or granule cell layer (panel B), for KChIP4 in the molecular layer (panel C) or granule cell layer (panel D), for Kv4.2 in the molecular layer (panel E) or granule cell layer (panel F) or for Kv4.3 in the molecular layer (panel G) or granule cell layer (panel H) was dramatically reduced (arrows) or mostly absent from the cerebellar cortex. Abbreviations: s, dendritic spines of Purkinje cells; pf, parallel fibre; D, dendrite of granule cells; mf, mossy fibre; GC, granule cell. Scale bars: A,B,D: 500 nm; C,E,G: 500 nm; F,H: 2 µm.
